# Meeting Report: The Institute for Genomic Research/Wellcome Trust Conference: Genomes 2004 14–17 April 2004, the Wellcome Trust Conference Centre, The Wellcome Trust Genome Campus, Hinxton, Cambridge, Uk

**DOI:** 10.1002/cfg.431

**Published:** 2004

**Authors:** Jo Wixon

**Affiliations:** MRC RFCGR Hinxton, Cambridge CB10 1SB United Kingdom

## Abstract

This conference brought the microbial genomics community together to share their most up-to-the-minute achievements, so much so that several talks cannot be covered here, as the work discussed has not yet been published. This meeting report has details of a cross-section of the talks from the sessions on ‘Genome analysis and comparative genomics’, ‘Computational genomics’ and ‘Functional genomics’, ranging from studies on complex environmental samples, to specific pathogenic bacteria, to yeasts.


**Comparative and Functional Genomics**
*Comp Funct Genom* 2004; **5**: 491–496.
Published online in Wiley InterScience (www.interscience.wiley.com). **DOI:** 10.1002/cfg.431

**Feature**

### Meeting Report: The Institute for Genomic Research/Wellcome Trust Conference: Genomes 2004 14–17 April 2004, The Wellcome Trust Conference Centre, The Wellcome Trust Genome Campus, Hinxton, Cambridge, UK

Jo Wixon*
*MRC RFCGR, Hinxton, Cambridge CB10 1SB, UK*

*Correspondence to:
Jo Wixon, MRC RFCGR, Hinxton, Cambridge CB10 1SB, UK.
E-mail: jwixon@rfcgr.mrc.ac.uk*

### Abstract

**This conference brought the microbial genomics community together to share their most up-to-the-minute achievements, so much so that several talks cannot be covered here, as the work discussed has not yet been published. This meeting report has details of a cross-section of the talks from the sessions on 'Genome analysis and comparative genomics', 'Computational genomics' and 'Functional genomics', ranging from studies on complex environmental samples, to specific pathogenic bacteria, to yeasts.** **Copyright © 2004 John Wiley & Sons, Ltd.**

### Presentations

The meeting was opened with a keynote lecture by **Philip Hugenholtz** (University of California, Berkeley, USA), who introduced the field of **sequencing environmental samples** to study bacterial populations and described a pilot project his group are working on, looking at acid mine drainage samples.

Only 1% of microbes can be grown on plates and these are clonal populations, rather than the complex communities in which microbes live. Taking a very challenging environment for microbes, with pH typically below 1, abundant toxic metals, no light and limited supplies of organic carbon and fixed nitrogen, they hoped to have a simple community on which to test shotgun sequencing of PCRs from environmental samples. Even in such harsh conditions, they initially found five bacteria, one archaean, and fungi and amoebi (they have done less work on the eukaryotes to date, but plan to investigate both these and the virus component of the community). The principal bacteria were *Leptospirillum* (iron oxidizers) and *Sulfobacillus*, accompanied by an iron oxidizing archaean, '*Ferroplasma*'.

Analysing their sequences, they found two GC content peaks, at 38% and 55%, identifying archaeal and bacterial sequences, respectively. The archaeal reads had three peaks of BLAST match scores, which correlated with *Ferroplasma* type I, type II, or G plasma. The bacterial reads could be separated into *Leptospirillum* type II and type III after binning contigs by read depth and GC content. The sequence data they have on these microbes have already yielded information on how they survive in such an inhospitable environment, and interesting observations on the evolution of their genomes.

They also found a few sequences from a euryarcheotan, which looks like a very odd archaean. This was typical of the community, which appears simple in terms of the dominant strains but very complex in terms of rare strains. It is clear that they

Copyright © 2004 John Wiley & Sons, Ltd.

need enrichment approaches if they are to detect these rare types.

### Genome analysis and comparative genomics

**Siv Andersson** (University of Uppsala, Sweden) presented the analysis of two ***Bartonella* genomes**, *henselae* and *quintana* [1]. The gene order of these two genomes is largely the same, with some islands that are different (prophage and integrase fragments), these being mainly found in intergenic regions. There is a significant proportion of non-coding sequence (26%) and pseudogenes are common (128 in *henselae*, 175 in *quintana*); several *quintana* pseudogenes are whole in *henselae*. Rearrangements between the two genomes are often flanked by insertion remnants, so it looks as though phage and plasmid insertions were common in their ancestor, with differential loss and rearrangement occurring post-divergence. *B. quintana* appears to represent a small subset of *B. henselae*, as *Rickettsia prowazekii* is to *R. conorii*.

Her group have made a microarray using human and feline strains of *Bartonella*, this has been used to confirm that *B. koehlerae* is intermediate between *henselae* and *quintana. B. koehlerae* shows some overlap in gene loss, giving an idea of the age of some events. The array has also been used to look at diversity in isolates of *henselae*, showing that there are no gene content differences between predominantly human or feline isolates; the specificity looks more likely to be due to copy number differences of the islands.

They have identified a set of *Bartonella*-specific genes, with no homology to other genes, or that look most like $\alpha$-proteobacterial extrachromosomal genes, which could imply that the ancestor of *Bartonella* had an extrachromosomal plasmid that was integrated into the genome at some point.

The Genolevures project [2] has used **yeast comparative genomics** to study the evolution of yeasts. **Bernard Dujon** (Institut Pasteur, France) explained that the team first sequenced 13 hemiascomycete yeasts to low coverage, discovering far more diversity than they had expected. Their comparison of these genomes has clarified the placement of the ancient whole genome duplication, as being present in *sensu stricto Saccharomyces* and *Candida glabrata*, but not in *Kluyveromyces*.

They have increased the coverage of the *sensu stricto Saccharomyces* and decided to complete *Candida glabrata, Debaryomyces hansenii, Kluyveromyces lactis* and *Yarrowia lipolytica*. They see much variation between these species in genome size and chromosome number (Table 1), and in rDNA locations and copy numbers.

**Table 1.** Yeast genomes fully sequenced by Genolevures

| Species | Genome size (Mb) | No. of chromosomes |
|---|---|---|
| *C. glabrata* | 12 | 13 |
| *K. lactis* | 10 | 6 |
| *D. hansenii* | 12 | 7 |
| *Y. lipolytica* | 20 | 6 |

They have identified 32 824 yeast proteins (including those from *S. cerevisiae*) and clustered them into 3410 robust families; ~2000 of the families are 'universal', with just more than half of these having only one orthologue per species.

Looking at the genome maps, there is no real synteny, with ~500 syntenic clusters in each case between *S. cerevisiae* and *C. glabrata, S. cerevisiae* and *K. lactis*, and *C. glabrata* and *K. lactis. D. hansenii* and *Y. lipolytica* have far fewer clusters of synteny, with far more rearrangement. Looking at the genomes, they can see that different modes of evolution have played a part at different points (Figure 1). Looking at the sequence similarity of genes found in pairs, they can see two populations, those with lower similarity, which are the product of the ancient whole genome duplication, and those with greater similarity, which are the result of more recent segmental duplications.

**Jacques Ravel** (The Institute for Genomic Research, USA) presented work on **comparative genomics of *Bacillus anthracis***. The team chose a diverse range of genomes, including the Ames genome, the Florida outbreak genome and a *B. cereus* isolate. On deciding to close their 12X drafts, they found that the same gaps were present in each genome, enabling them to use the same PCR primers to cross them in each sample.

They looked in particular at a *B. cereus* isolate (*B. cereus* G9241) that caused an anthrax-like disease. Biochemical and phenotypic tests classify it as a *cereus*, not an *anthracis*, but when they compared its sequence to their other genomes, they saw that the distance between it and *cereus* was very similar to that between it and *anthracis* [4].

They have found that G9241 has a plasmid that has 99.6% similarity to the *anthracis* plasmid pX01

Copyright © 2004 John Wiley & Sons, Ltd. 

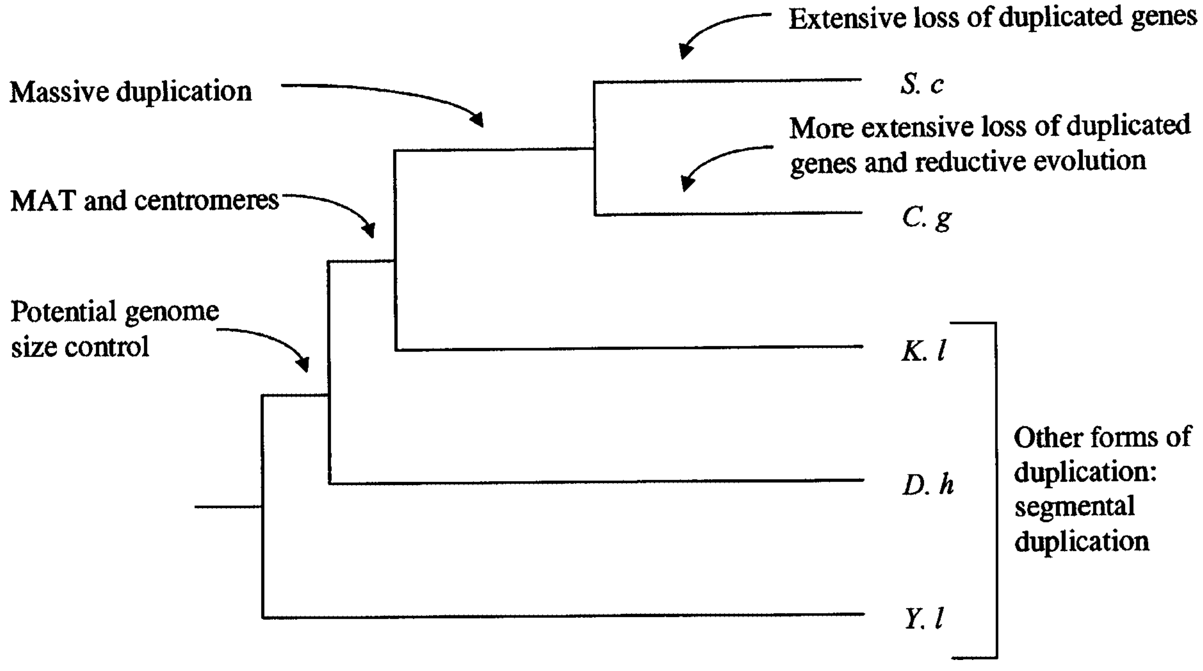

**Figure 1.** Modes of yeast genome evolution. S. c, *Saccharomyces cerevisiae*; C. g, *Candida glabrata*; D. h, *Debaryomyces hansenii*; K. l, *Kluyveromyces lactis*; and Y. l, *Yarrowia lipolytica*

(which encodes the lethal toxin complex) but there is no plasmid with homology to the *anthracis* pX02 (capsule-encoding) plasmid; instead, it has a novel plasmid that encodes a capsule that is novel, but like that of *anthracis*.

### Computational genomics

The numbers of bacterial and eukaryotic RNA genes are growing, with many found in the last two years. **Alex Bateman** (The Sanger Institute, UK) described the Rfam RNA gene prediction tool and **RNA gene family database** [8] that are very similar in design and style to Pfam [7], the well-known protein resources provided by his group. The prediction tool aims to predict all types of non-coding RNA (ncRNA) genes in an automated fashion. It is comprehensive and works well for prokaryotes but, as yet, is slow and memory-hungry and will need to be made more sensitive to work well for eukaryotes. RNA gene prediction is very hard, because the sequence is not conserved; it is the base pairing in hairpins that is conserved.

The Rfam5 database has 176 ncRNA families, annotating more than 100 000 sites in Ensembl (Rfam6 will have 300 ncRNA families). Family pages include secondary structure annotation, multiple sequence alignments and organism distribution information. It is also possible to view the predictions for whole genomes, e.g. *Escherichia coli* has 152 predicted genes and 47 families; it takes the tool about a day to predict for a whole bacterial genome. The database also has data on *cis*-regulatory sequences, such as temperature-sensitive hairpins in mRNAs.

Moving neatly on to **annotation of microbial proteins, Amos Bairoch** (Swiss Institute of Bioinformatics, Switzerland) gave the audience an update on the status of microbial sequence datasets and on the HAMAP (High-quality Automated and Manual Annotation of Microbial Proteomes [3]) resource that he manages. Vast amounts of microbial sequence data are being generated, with microbial proteomes set to rise from making up 30% of Swiss-Prot [10] to 50%, with entries from 132 microbes (116 bacteria and 16 Archaea). However, the trend for not finishing genomes is causing a lack of first-pass annotation and key supporting data, and incomplete datasets in terms of gene content.

In the case of HAMAP, when a new genome comes in, similarity searches are performed to identify which proteins belong to well-defined families, which may belong to families and which

Copyright © 2004 John Wiley & Sons, Ltd.

have no match (orphans). Orphans with no BLAST match or InterPro hit undergo a range of analyses, including signal sequence detection and coiled-coil prediction, in the hope of finding any clues at all about their function. The team are developing rules to assign proteins to well-known families, or to well defined but uncharacterized families (UPFs), which allow this process to be automated. Stringent cut-off criteria are used, as the group prefer to miss a member rather than risk assigning a false member, and all families are mapped to GO terms. 1031 families cover 41 158 Swiss-Prot entries, and 10–50% of any given microbial proteome can be annotated to Swiss-Prot level quality.

They plan to make new families for genes with limited taxonomic range and to refine their complex families, as these still need much work by an annotator after the initial match is made. They have defined rules for their 270 UPFs, which help to reduce the propagation of erroneous annotation, when no experimental data is available to back up the inferences drawn.

Proteomics data has its own problem, in that it is common for users to take the top hit from mass spectrometry size match data, which is often wrong. It has proved very difficult for the team to work on these cases. He also included a plea to remember about inteins, selenocysteines, pyrrolysines and ribosomal frameshifting, all of which can greatly complicate annotation.

**Ross Overbeek** (Fellowship for Interpretation of Genomes) abruptly changed tack by asking the audience to consider **annotation of subsystems** (such as a biosynthetic pathway) rather than of organisms, or genomes. Putting it another way, he described this as expert annotation, rather than the work of an annotation expert. He feels that noting the clustering of genes from a pathway has a dramatic effect on accurate gene prediction. He has assembled several examples of cases where an unassigned gene in an operon could be predicted to perform the step that was missing an enzyme from the pathway. He also asserts that this approach can be used to disambiguate paralogues of a gene, with the copy residing in the operon being the one acting in the pathway. Once the correct identity of one of these genes has been uncovered, then it can be assigned in multiple species.

He estimates that 100–300 subsystems would cover the core machinery of the cell, but would start with just a few to demonstrate the validity of the approach. Once subsystems are annotated clearly, it should be possible to generate pipelines for automation. He feels that gene proximity has been used somewhat clumsily so far, and that this has only worked because the signal is so strong. He wants to try applying statistics to the analysis to achieve a more rigorous approach.

He also briefly mentioned the SEED [11], an annotation framework provided by the Fellowship for Interpretation of Genomes. This open-source system (the work of four collaborating institutes) allows person-to-person exchange of subsystems or annotation.

### Functional genomics

The KEGG (Kyoto Encyclopedia of Genes and Genomes) database [5] has long been a useful resource of information on biochemical pathways and the genes, proteins and ligands involved in those pathways. **Minoru Kanehisa** (Kyoto University, Japan) described the **expansion of KEGG to include chemical knowledge** relating to biochemical reactions. The KEGG LIGAND database contains 10 882 COMPOUND entries and 10 420 GLYCAN entries (added last year); 114 PEPTIDE entities are to be added this year. It has 5961 chemical reactions (REACTION), 8605 reactant pairs and around 1000 reaction classifications. The resource includes chemical structures, which can be compared to find locally similar structures.

The team are developing a reaction classification (RC) that can be assigned to given pairs of compounds. The RC numbers consist of three numbers; the first position represents the reaction centre, the second the difference region and the third the matched region. From 3253 EC numbers, they have 5227 reactions and 8605 reactant pairs, these can be assigned to 1018 RC numbers.

Their RC numbers can be used to work out up to the third position of EC number in ~90% of cases. For an unassigned reaction, they can use the structure of the reactant or product to assign their RC number and use that to work out most of the EC number. The substrate is then used to work out the fourth position of the EC number. They have been using this knowledge to predict the EC numbers of missing enzymes in biochemical pathways, concentrating in particular on *Arabidopsis* metabolism.

Copyright © 2004 John Wiley & Sons, Ltd.

**George Weinstock** (Baylor College of Medicine, USA) presented the results of **functional genomics studies of spirochaetes**, with the majority of the work focusing on *Treponema pallidum*, the causative agent of syphilis. The first part of the work was a project to clone and express all of the genes from this bacterium in *E. coli* and test for antigenicity [6]. This approach identified 106 proteins, including 21 of the 28 previously known antigens; 48% of these proteins have leader sequences (compared to 24% in the whole gene set) and 34 of the antigens showed specificity for human, most of which showed antigenicity in the rabbit. Time-course experiments were used in the human and rabbit antigenicity tests, uncovering early and late antigens in each case. They have also made microarrays of the clone set, and have shown good correlation with RT-PCR data and proteomics data.

Genomic comparisons with *T. pertenue* (which causes yaws) and *T. paraluiscuniculi* (which causes rabbit syphilis) show that there are very few differences between *T. pallidum* and the yaws form, but about 10-fold more variation between the human and rabbit syphilis-causing strains. He also discussed the recently completed genome sequence of *T. denticola* [9]. This has 825 genes in common with *T. pallidum* and 335 genes in common with the other three sequenced spirochaetes, although none of these is spirochaete-specific. Its genome is completely reorganized with respect to the gene order in *T. pallidum* and it has many more ABC efflux pumps than other bacteria.

*Rhodococcus* is a soil actinomycete with potential applications in bioremediation [it is among the best polychlorinated biphenyl (PCB) degraders] and green chemistry. **William Mohn** (University of British Columbia, Canada) described studies of the complex system for **aromatic compound degradation in *Rhodococcus***. The bacterium has streamlined and convergent pathways for benzoate and phthalate degradation, encoded by genes on two plasmids (pRHL1 and 2) and its chromosome. His group used proteomic analyses to identify the genes involved, showing induction of ∼80% of those genes that had been predicted to have a role and that the majority of these were barely expressed in bacteria grown on pyruvate.

While the degradation of benzoate and phthalate result in the formation of a common enol lactone intermediate by different routes, their analyses have shown that this is converted to TCA cycle intermediates by the same route in each case, there being only one pathway for this.

Biphenyl and PCB degradation result in the formation of benzoate and a non-aromatic intermediate. There are multiple potential systems for each step in this process, so the team aimed to determine which genes did which step, or whether it was a redundant process. Using an array with ∼2500 probes, they compared biphenyl, ethylbenzene, or isopropylbenzene with pyruvate-grown cells. Some genes were upregulated on only one of the pollutants and 112 on all three, indicating that there is a suite of enzymes involved in the degradation of all three compounds. This redundancy could produce a more robust system with specificity for a wider range of analogues, both of which are features that would be advantageous to *Rhodococcus*.

### Conclusion

This was a successful, well-attended conference that several groups chose as the venue to break new stories coming out of their work. A wide range of microbial interests was represented, including archaea, soil bacteria, animal pathogens, yeasts and filamentous fungi. The talks provided an impressive showcase of the excellence and breadth of work that is going on in microbial genomics.

The conference was held at the Wellcome Trust Conference Centre [12], on the Wellcome Trust Genome Campus in Hinxton. The combination of excellent facilities with beautiful surrounding grounds provided an ideal atmosphere for discussions after the sessions and the proximity of the neighbouring institutes allowed delegates and Genome Campus staff to meet and exchange ideas.

### References

1. Alsmark CM, Frank AC, Karlberg EO, *et al*. 2004. The louse-borne human pathogen *Bartonella quintana* is a genomic derivative of the zoonotic agent *Bartonella henselae*. *Proc Natl Acad Sci USA* **101**(26): 9716–9721.
2. Genolevures: **http://cbi.labri.fr/Genolevures/**
3. HAMAP: **http://www.expasy.org/sprot/hamap/**
4. Hoffmaster AR, Ravel J, Rasko DA, *et al*. 2004. Identification of anthrax toxin genes in a *Bacillus cereus* associated with an illness resembling inhalation anthrax. *Proc Natl Acad Sci USA* **101**(22): 8449–8454.
5. KEGG: **http://www.genome.jp/kegg/**

Copyright © 2004 John Wiley & Sons, Ltd. 

6. McKevitt M, Patel K, Smajs D, *et al*. 2003. Systematic cloning of *Treponema pallidum* open reading frames for protein expression and antigen discovery. *Genome Res* **13**(7): 1665–1674.
7. Pfam: **http://www.sanger.ac.uk/Software/Pfam/**
8. Rfam: **http://www.sanger.ac.uk/Software/Rfam/**
9. Seshadri R, Myers GS, Tettelin H, *et al*. 2004. Comparison of the genome of the oral pathogen *Treponema denticola* with other spirochaete genomes. *Proc Natl Acad Sci USA* **101**(15): 5646–5651.
10. Swiss-Prot: **http://www.expasy.org/sprot/**
11. The SEED: **http://theseed.uchicago.edu/FIG/index.cgi**
12. Wellcome Trust Conference Centre: **http://www.wtconference.org.uk**

Copyright © 2004 John Wiley & Sons, Ltd. 
